# Surgeon handedness affects the acetabular cup positioning during primary total hip arthroplasty regardless of the surgical approach. a systematic review and metanalysis

**DOI:** 10.1186/s12891-024-07868-2

**Published:** 2024-10-07

**Authors:** Ahmed A. Khalifa, Ahmed Abdelazim Hassan

**Affiliations:** 1 Orthopedic Department, Qena Faculty of Medicine, South Valley University, Qena, Egypt; 2 Orthopaedic and Traumatology Department, Assiut University School of Medicine, Assuit, 71515 Egypt

**Keywords:** Total hip arthroplasty, Total hip replacement, Handedness, Inclination, Anteversion, Acetabular cup position, Systematic review

## Abstract

**Purpose:**

The aim was to investigate the effect of surgeon handedness on acetabular cup positioning, functional outcomes, and dislocation incidence during primary THA.

**Methods:**

A systematic review was conducted according to the PRISMA guidelines. Studies published in English were searched in three databases (PubMed, Embase, and Scopus). A dominant side is a right-handed (RHD) or left-handed (LHD) surgeon who operates on the right or left hip, respectively. The opposite is considered to be the non-dominant side. We used odds ratios for dichotomous data and mean differences for continuous data, with 95% confidence intervals for quantitative data synthesis. Heterogeneity was assessed using the I² test, with outcomes graphically represented in a forest plot and a p-value of < 0.05 considered statistically significant; analyses were performed using Review Manager 5.4 (RevMan 5.4.1). >.

**Result:**

Four observational studies were included out of 98 articles. Ten experienced surgeons participated (8 RHD and 2 LHD) and operated on 822 patients (1484 hips), divided equally between dominant and non-dominant sides, and the posterolateral approach was utilized in 80.9% of THAs. RHD surgeons operated on 1404 (94.6%) THAs. The pooled synthesis for inclination indicated no significant difference between either side [MD: 0.10 (95% CI -2.10 to 2.30, *P* = 0.93, I² = 91%)]. While the difference was significant for anteversion [MD: -2.37 (95% CI -3.82 to -0.93, *P* = 0.001, I² = 31%)]. The functional outcome was better on the dominant side [MD: 1.44 (95% CI 0.41 to 2.48, *P* = 0.006, I² = 0%)], and the dislocation incidence was significantly higher on the non-dominant side [OR: 0.45 (95% CI 0.25 to 0.81, *P* = 0.008, I² = 0%)].

**Conclusion:**

Surgeon handedness and whether operating on the dominant or non-dominant side could affect the acetabular cup positioning and outcomes during primary THAs, even in the hands of high-volume surgeons.

**Supplementary Information:**

The online version contains supplementary material available at 10.1186/s12891-024-07868-2.

## Introduction

Surgeon handedness and its effect on various aspects of training and practice have been a concern of surgeons among different specialties [1–5]. Nevertheless, for orthoapedic surgeons operating on skeletal areas, mostly presented bilaterally, the orientation and degree of comfort differ for right- or left-handed surgeons according to the operating side [1, 6, 7]. Such effect was attributed to various factors, including but not limited to a more powerful dominant limb with subsequent better control of skilled activities, refined and more accurate motor control, faster manipulation, longer time to fatigue, and better ability of spatial orientation [8–13].

Sabharwal et al. reported that 46% of the left-handed (LHD) participants in their study reported difficulties in handling right-handed (RHD) instruments; furthermore, LHD trainees reported difficulties while training by RHD teaching surgeons, and these difficulties were significantly greater than their RHD peers’ trainees, 36% and 61%, respectively (*p* < 0.001) [1].

Concerning orthopedic procedures, Moloney et al. attributed increased sliding hip screw failures while treating left hip peri-trochanteric fractures to screw malpositioning indued by RHD surgeons [14]. Additionally, Liu et al. showed significantly higher femoral implant malpositioning in the sagittal plane during primary total knee arthroplasty (TKA) when an RHD surgeon operated on the non-dominant compared to the dominant side [7]. Furthermore, Mehta and Lotke reported that the knee functional and pain outcomes were significantly better at six-month and one-year follow up on the dominant (377 TKAs) compared to the non-dominant (351 TKAs) side when an RHD surgeon performed surgeries [6].

Proper implant positioning during total hip arthroplasty (THA) is paramount to achieve better outcomes, including long-term survival [15–18], and factors affecting acetabular cup placement were thoroughly reported in the literature, including but not limited to patient’s position, whether lateral or supine [19, 20], which surgical approach [21, 22], surgeon experience and learning curve [23–25], pelvic tilt and spinopelvic relationship [26, 27], and patient obesity [28], and in most of the cases it could be multifactorial [18, 23, 24, 27–32]. One factor that has yet to be deeply investigated is the probable effect of surgeon handedness.

So, the primary objective of the current systematic review was to investigate the effect of surgeon handedness on acetabular cup positioning during primary THA. The secondary objectives were to compare the functional outcomes and dislocation incidence between the dominant and non-dominant sides.

## Methods

We conducted the current systematic review according to the Preferred Reporting Items for Systematic Reviews and Meta-Analyses PRISMA guidelines (Supplementary file 1) [33]. The protocol was registered in PROSPERO (CRD42023442797).

### Eligibility criteria and search strategy

Comparative studies (randomized controlled trials (RCTs), cohort studies, and case-control studies) reported in English discussing the effect of surgeon handedness on acetabular cup placement during primary total hip arthroplasty where the acetabular cup was placed using manual instruments were included. While reports in other languages, review articles, technical notes, cadaveric studies, and case reports were excluded.

In May 2023, we systematically searched the English language literature published in the past 20 years in PubMed, Embase, and Scopus databases using a combination of the following terms: “Total hip/handedness” and their synonyms using a boolean operator, which must be included in the title and abstract. Furthermore, a manual secondary search of the bibliography of the included articles’ full text for possible related articles was conducted (the detailed search strategy is shown in supplementary file 2).

### Study selection

All the identified articles were downloaded to the EndNote 20 program, followed by the title and abstract screening for eligibility independently by two authors. After finalizing the selection process, a discussion between the authors was needed to resolve any controversy and settle on the final articles that were included. Furthermore, the reference lists of the articles that were finally included were checked, and publications citing the included articles were evaluated for possible eligible articles.

### Data collection and extraction

Data from the included articles were extracted independently by the authors into a predetermined Excel sheet, which will include article characteristics (year of publication, country of origin, author name, and study type), Population characteristics (demographics of the included patients, type of THA, and management details), and outcomes (acetabular cup position (inclination, anteversion, and percentages located within the safe zone), functional or clinical outcomes, and complications). The established terminology through the review was to report a dominant side when an RHD surgeon operated on the right hip or an LHD surgeon operated on the left hip; the opposite is considered the non-dominant side.

### Risk of bias assessment

Since no RCTs were identified, the National Institutes of Health (NIH) quality assessment tool for observational cohort and cross-sectional studies was used for assessment [34], carried out independently by the authors; both reports were compared together, and agreement was resolved by discussion between the authors.

### Data analysis and synthesis

#### Qualitative synthesis

Data extracted from the included articles was presented in tables documenting the basic characteristics of the included studies and the outcomes.

#### Quantitative synthesis

The odds ratio (OR) was used to express the dichotomous data, and the mean difference (MD) was used for continuous data to express the measure’s effect with a 95% confidence interval. The I^2^ and Chi² tests were utilized to quantify the inconsistency and evaluate whether the observed differences in study results are due to heterogeneity rather than random chance or sampling error. A random-effects model was used to ensure that the results reflect both within-study and between-study variability, helping in achieving more robust and generalizable conclusions. A forest plot was used to graphically represent the differences in outcomes in the two treatment groups. A p-value of < 0.05 will be considered statistically significant. Statistical analysis was performed by Review Manager 5.4 (RevMan 5.4.1).

## Results

### Search results

**Fig. 1:**
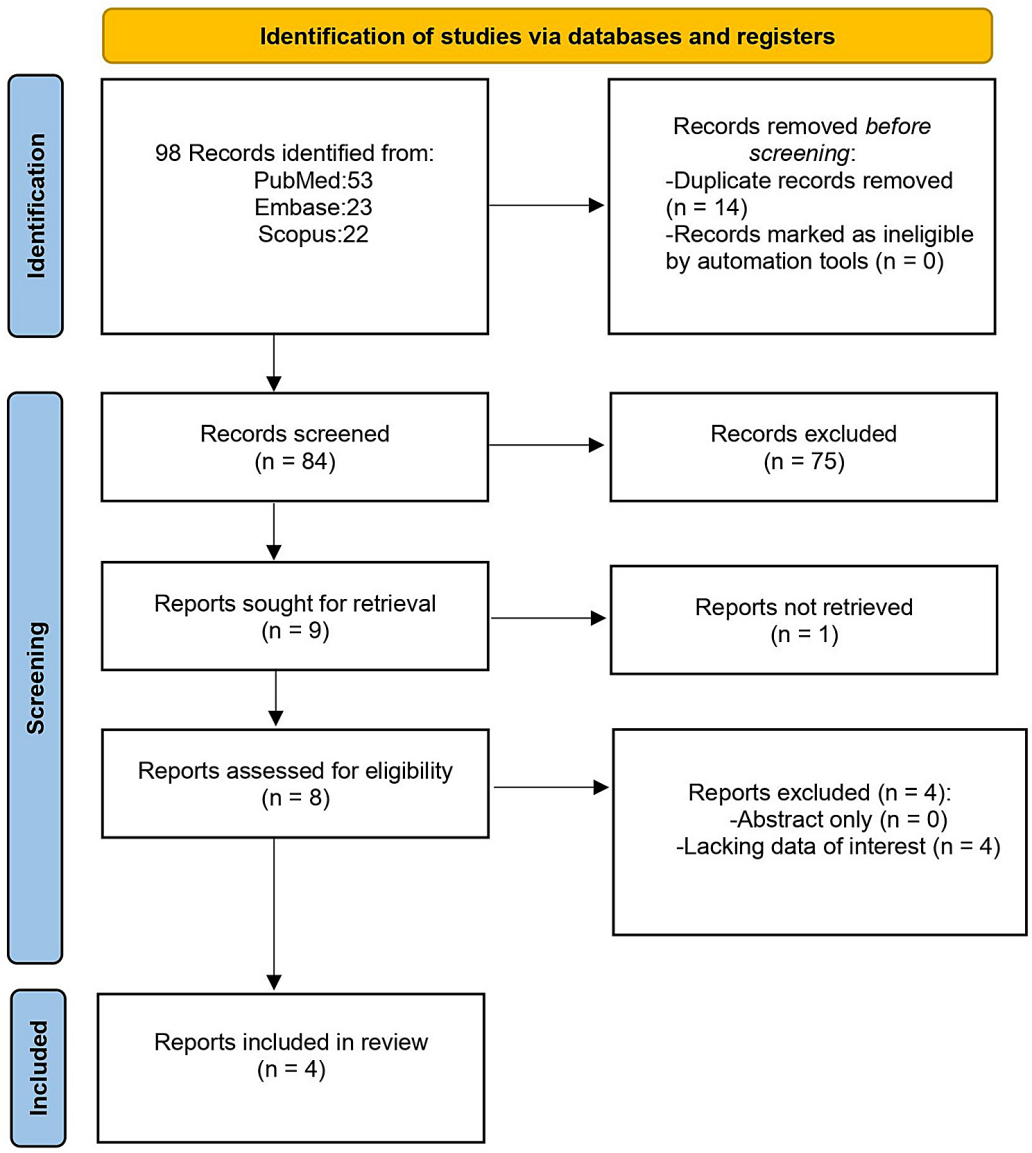
Flowchart showing the study selection process according to the Preferred Reporting Items for Systematic Reviews and Meta-Analysis (PRISMA)

The initial search of the three databases revealed 98 articles, of which, after evaluation, four articles were eligible for inclusion [35–38]. Search strategy details are shown in the PRISMA flow chart (Fig. 1).

### Characteristics and quality of the included studies (Table 1)

**Table 1:** Basic characteristics of the included studies

Article	Year	country	Number of Patients	Number of THAs	Age*	Gender M/F	BMI*	Diagnosis** (% patients)	Number of Surgeons	Handedness	Surgical Approach	Type of acetabular cup fixation	Mean operative time (Min.)	
													Dominant	Non-dominant
Pennington et al.	2014	UK	160	160	NR	NR	NR	OA (100%)	4	2 RHD, 2LHD	-PLA (2 surgeons, one RHD and one LHD). -DLA (2 surgeons, one RHD and one LHD)	NR	NR	
Song et al.	2018	China	498	996	45.8 ± 13 (20–83)	307/191	24.4 ± 3.95 (13.2–44.8)	ONFH (48.2%) OA (22.5%) AS (21.5%) DDH (4.8%) RA (3.0%)	3	RHD	PLA	cementless	83.8	85.8
Kong et al. (PLA)	2020	China	62	124	40.18 ± 11 (21–67)	38/24	21.9 ± 2.89 (17.93–29.21)	ONFH 67.74%, DDH 25.81%, Others 6.45%	2	RHD	PLA	cementless	NR	
Kong et al. (DAA)	2020	China	102	204	43.39 ± 12	59/43	22.7 ± 2.79	ONFH 94.12%, DDH 2.94%, RA 2.94%	1	RHD	DAA	cementless	NR	

*Data presented as Mean ± SD (range). **Data presented as percentages. PLA: posterolateral approach, DAA: direct anterior approach, UK: United Kingdom, M: male, F: female, NR: not reported, BMI: body mass index, OA: osteoarthritis, ONFH: osteonecrosis of the femoral head, AS: ankylosing spondylitis, DDH: developmental dysplasia of the hip, RA: rheumatoid arthritis, RHD: right-handed, LHD: left-handed, DLA: direct lateral approach, Min.: minutes

The four included studies were retrospective observational studies, with ten surgeons participating (8 RHD and 2 LHD), and all were high-volume experienced surgeons (as reported by the authors in all articles). They operated on 822 patients (1484 hips); all were primary THAs where the acetabular cup was placed using manual instruments and was divided equally between dominant and non-dominant sides. 1200 (80.9%) THAs were performed through the posterolateral approach (PLA), 204 (13.7%) through a direct anterior approach (DAA), and 80 (5.4%) through a direct lateral approach (DLA). RHD surgeons operated on 1404 (94.6%) THAs, while 80 (5.4%) THAs (40 THA through the PLA and 40 THAs through the DLA) were operated on by LHD surgeons. All patients were operated upon in the lateral decubitus position, except for patients in the study by Kong et al. (DAA) [38], who were operated upon in a supine position. All surgeons stand on the same side of surgery.

Although all the articles indicated that the acetabular cups were placed using manual techniques, none explained the exact technique to obtain the desired intraoperative cup position.

Regarding the targeted zone for cup placement, three articles clearly stated their desired targets, upon which the acetabular cup positioning accuracy was evaluated postoperatively. Two articles [36, 38], defined the target zone as the safe zone described by Lewinnek et al. (an anteversion of 15° ± 10° and an inclination of 40° ± 10°) [30]. In the third article [37], the authors narrowed the target zone to 20° ± 5° of anteversion and 40° ± 5° of inclination.

The quality of all studies was graded as fair per the NIH quality assessment tool (Supplementary file 2).

### Outcomes (Table 2)

**Table 2:** Primary and secondary outcomes of the included studies

Variables			Study			
Parameter	side		Pennington et al.	Song et al.	Kong et al. (PLA)	Kong et al. (DAA)
Acetabular cup positioning						
Cup inclination*	Dominant		46.4 ± 4.31	38.59 ± 6.84 (18–72.5)	39.35 ± 5.26 (23–48)	39.42 ± 7.19
	Non-dominant		43.5 ± 5.22	37.5 ± 6.76 (15.5–70)	40.35 ± 5.77 (25–55)	42.61 ± 7.32
Cup anteversion*	Dominant		NR	22.01 ± 6.35 (7.5–41.5)	22.44 ± 8.67 (1–41)	15.79 ± 6.99
	Non-dominant			25.28 ± 7.16 (6.5–45)	24.77 ± 10.44 (0–55)	16.91 ± 7.49
Located within safe zone (% of cups) according to Lewinnek et al.**	Dominant	Inclination:	100%	88%	NR	NR
		Anteversion	NR	71%		
		both	NR	62%	32.25%	81.37%
	Non-dominant	Inclination:	100%	87%	NR	NR
		Anteversion	NR	52%		
		both	NR	46%	27.41%	73.53%
The mean difference between both sides*	Inclination		3 degrees	1.08 ± 9.46 (− 47–26)	Difference > 5 degrees in 77%	NR
	Anteversion		NR	3.27 ± 7.37 (− 17.5–24.5)		
LLD (mm)*	Dominant		0.5 ± 5.34	NR	NR	NR
	Non-dominant		0.0 ± 5.67			
Center of Rotation Medialization*	Dominant		0.4 ± 2.04	NR	NR	NR
	Non-dominant		1.7 ± 4.54			
Functional outcomes						
Follow up period (months)			NR	NR	3 months	17.11 ± 2.58
HHS*	Dominant		NR	NR	83.63 ± 9.02 (71–95)	94.33 ± 4
	Non-dominant				81.11 ± 9.3 (68–95)	93.01 ± 3.94
Complications						
Dislocation**	Dominant		NR	16 (3.21%)	0 (0.0%)	1 (0.98%)
	Non-dominant			35 (7.02%)	2 (0.0%)	0 (0.0%)
PJI**	Dominant			NR	0 (0.0%)	NR
	Non-dominant				1 (1.6%)	
vascular injury**	Dominant				NR	0 (0.0%)
	Non-dominant					2 (1.96%)
periprosthetic fracture**	Dominant					3 (2.94%)
	Non-dominant					1 (0.98%)
LFCN palsy**	Dominant					9 (8.82%)
	Non-dominant					12 (11.76%)
HO**	Dominant					2 (1.96%)
	Non- dominant					0 (0.0%)

*Data presented as Mean ± SD (range). **Data presented as percentages. LLD: leg length difference, PJI: periprosthetic joint infection, LFCN: lateral femoral cutaneous nerve, HO: heterotopic ossification, NR: not reported, PLA: posterolateral approach, DAA: direct anterior approach

#### Acetabular cup positioning

##### Inclination

**Fig. 2:**
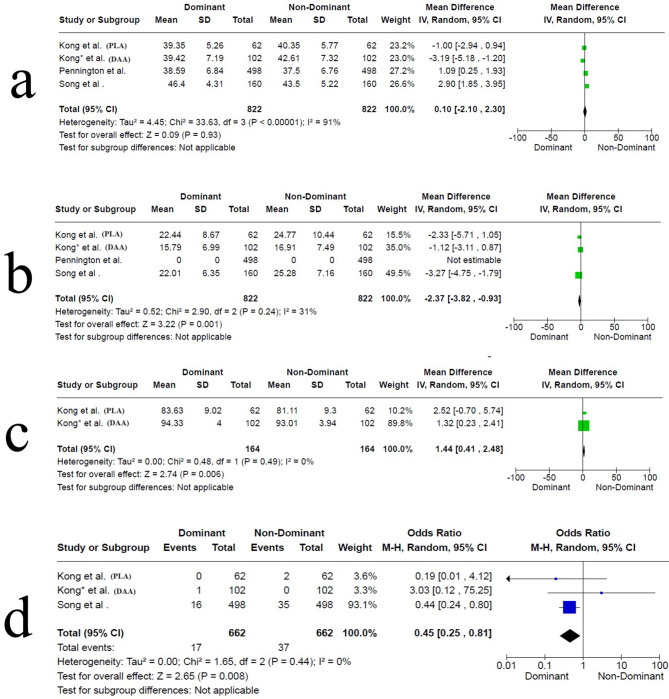
Forest plot showing the pooled results of the included studies. a, acetabular cup inclination. b, acetabular cup anteversion. c, functional outcome according to Harris Hip Score. d, dislocation rate. (Forest plot a, shows high heterogeneity (I² = 91%), indicating substantial variability among the studies. Forest plot b, demonstrates moderate heterogeneity (I² = 31%), suggesting that the observed differences are partially due to true heterogeneity. In both, a random-effects model accounted for the variability and provided a more conservative estimate of the overall effect. Forest plots c and d, show no heterogeneity (I² = 0%), implying minimal variability among the studies. In such cases, a fixed-effects model could be justified; however, a random-effects model was still applied to provide a more generalized result, consistency across analyses, and account for potential unobserved heterogeneity)

This item was reported in all studies; three studies showed a significant difference between both sides (in two studies, Pennington et al. [35] and Song et al. [36], the dominant side cup inclination was higher than the non-dominant side. In contrast, in the study by Kong et al. (DAA) [38], the non-dominant side had higher cup inclination. The study by Kong et al. (PLA) [37], showed no statistically significant difference between both sides. The pooled synthesis indicated no significant difference between either side. The MD was 0.10 (95% CI -2.10 to 2.30, *P* = 0.93, I² = 91%), Fig. 2a.

##### Anteversion

It was reported in three studies [36–38]; cup anteversion was higher on the dominant side in the study by Song et al. [36], higher on the non-dominant side in the study by Kong et al. (PLA) [37], and not different between both sides in the study by Kong et al. (DAA) [38]. The pooled synthesis indicated a significant difference between both sides. The MD was − 2.37 (95% CI -3.82 to -0.93, *P* = 0.001, I² = 31%), Fig. 2b.

##### Percentage of cups located within the safe or targeted zones

This was reported in all studies. Pennington et al. [35] reported that 100% of the cups on both sides were within the Lewinnek safe zone for inclination. In the Song et al. study [36], the authors reported no difference in the percentage of cups located within the safe zone of inclination only, while the difference was significant regarding the anteversion and both parameters, being better on the dominant side. Kong et al. (PLA) [37] (who used a narrower target zone) reported no difference between both sides regarding the percentage of cups located within their target zone. Kong et al. (DAA) [38] reported no difference between both sides in the percentage of cups located within the safe zone for both inclination and anteversion.

##### Restoration of the hip center of rotation (COR)

This was reported in one study [35], where the authors reported less medialization on the dominant side; however, the differences did not reach statistical significance.

#### Operative time

This was only reported by Song et al. [36], where the authors showed that operative time was significantly longer on the non-dominant side.

#### Functional outcomes

Hip functional outcomes measured by Harris Hip Score (HHS) were reported in two studies [37, 38], and both reported no difference between both sides. In the Kong et al. (PLA) [37] study, HHS was reported postoperatively, while in the Kong et al. (DAA) [38] study, HHS was reported after a mean follow up of 17.11 ± 2.58 months. The pooled synthesis indicated a significant difference between both sides (favoring the dominant side). The MD was 1.44 (95% CI 0.41 to 2.48, *P* = 0.006, I² = 0%), Fig. 2c.

#### Complications

All complications reported in the respective studies are reported in (Table 2); however, we have to emphasize the instability issues (mainly dislocation) related to the acetabular cup positioning. Dislocation incidents were reported in three studies [36–38]; overall, it was more on the non-dominant side than the dominant side (37 vs. 17); however, only in the Song et al. study did the authors report that this difference reached statistical significance [36]. The pooled synthesis for dislocation incidence indicated a significant difference between both sides, with a higher incidence on the non-dominant side. The OR was 0.45 (95% CI 0.25 to 0.81, *P* = 0.008, I² = 0%), Fig. 2d.

## Discussion

Although it is considered one of the most successful surgical procedures [39], failures after THA still occur, partially attributed to component malpositioning [16, 17, 40]. So, it becomes evident that proper implant positioning during THA is crucial for better outcomes, including higher survival rates [15–18]; furthermore, how to get the component in the proper position, even without the assistance of newer technologies, is still a concern and was thoroughly reported in a recent review by Meermans et al. [18].

Various factors have been investigated to affect implant positioning, particularly the acetabular cup [18, 23, 24, 27–29, 31, 32]. However, the effect of surgeon handedness was studied less than other factors [35–38, 41]. The results of the current review indicate that surgeon handedness affects acetabular cup positioning (anteversion more than inclination) during primary THAs concerning which side is being operated (dominant vs. non-dominant) and a possible effect on the functional outcomes and dislocation rates. Although some of the reported differences could be considered clinically insignificant, the results should be interpreted considering that all the operated surgeons were “experienced” and operated on considerably straightforward primary THAs.

Possible reasons for differences in parameters between dominant and non-dominant sides based on surgeon handedness could be suggested.

### First, the side where the surgeon stands

In the current review, all surgeons reported standing on the same side of the surgery, which most surgeons commonly practice. So, an RHD surgeon operating on the right hip (dominant side) will prepare the acetabulum using mainly the right hand (holding the reamer or hammering the cup in place), and the left hand will support the instrument. In contrast, when operating on the left side (non-dominant side), the surgeon has to choose between leading the acetabulum preparation by the non-dominant hand (the left) or using the dominant hand, but in the latter, the body will be in a disadvantageous position [36].

Song et al. reported that while impacting a cementless acetabular cup on the non-dominant side while the patient is in the lateral decubitus position, the surgeons need to place their dominant hand (which is holding the hammer) above the non-dominant hand (which is holding the acetabular cup handle), which could affect the working space compared to operating on the dominant side, leading to an uncomfortable operating position. Furthermore, the authors attributed less operative time on the dominant side to comfortability during surgery [36].

However, if a surgeon chose to stand on the opposite side [40], this could seriously affect cup positioning, as shown in a study by Grammatopoulos et al. on a pelvic model aiming at assessing the accuracy of cup placement by surgeons of different experience levels and if standing on different sides of the operative table will affect cup positioning, the authors reported higher variability of cup inclination estimation (up to 14 degrees) when the surgeons changed his position to stand on the assistant side [42].

### Second, using manual instruments

Using manual instruments and freehand technique for acetabular cup placement, combining external and anatomical landmarks has been the standard for many surgeons, mainly if they cannot utilize newer technologies such as computer navigation or robotic-assisted surgeries [40, 43, 44]. Callanan et al. reported on the variability of freehand acetabular cup positioning when operating through various surgical approaches, where 57.3%, 37%, and 32% were within Lewinnek et al. safe zone when operating through the posterolateral, anterolateral, and direct lateral approaches respectively [31]. Furthermore, higher accuracy and consistency and fewer outliers of acetabular cup placement were proved when computer navigation assisted surgery compared to manual freehand techniques [45, 46].

All acetabular cups included in the current review were placed using the freehand technique. However, in one of the studies by Kong et al. (PLA) [37], the authors included a comparative group of 53 bilateral robotic-assisted THAs, where no difference in acetabular cup positioning between both sides was reported in this group; furthermore, the cups located outside the safe zone was lower than the manual freehand group (48% vs. 70%, *p* = 0.001), and the consistency (measured as > 5 degrees difference between both sides) was lower in the robotic-assisted THAs group (45% vs. 77%, *p* = 0.000).

### Third, surgeons experience

In the current review, while all the operating surgeons were considered “high-volume and experienced,” there are still differences in acetabular cup positioning between both sides. Barrack et al. reported that low-volume surgeons had a higher possibility of acetabular cup malpositioning [47]. Furthermore, less experienced surgeons showed less consistency for acetabular cup placement than experienced surgeons, as shown by Kim et al., where the authors found that hip COR restoration was significantly accurate and consistent in the hands of experienced surgeons [15]. Moreover, Bosker et al. reported that senior surgeons were better than residents regarding acetabular cup inclination and anteversion [23]. This raises concerns about amplifying differences reported in acetabular cup positioning when less experienced surgeons are involved.

### Fourth, the surgical approach and patient position

The surgical approach could play a role; variations in acetabular cup positioning were reported in the current review, where surgeries were performed through three main approaches, namely the DLA, PLA (patients were in the lateral decubitus position), and DAA (patients were in supine position). Crawford et al. reported differences attributed to the surgical approach when evaluating THAs performed by a single RHD surgeon; they found that cup positioning was generally better (according to cups located within Lewinnek safe zone) while utilizing DAA (supine position) compared to the DLA (lateral decubitus position); furthermore, the inclination was significantly higher on the dominant side for DAA (*p* = 0.03), while the anteversion was significantly higher on the dominant side for the direct lateral approach (*p* = 0.004) [41].

### Last, instruments control and spatial cognition

The ability to control the instruments better, as the dominant hand is more powerful than the non-dominant hand, enables the surgeon to have better control over skilled motor activities, faster manipulation, and longer time to fatigue [8, 12]. Furthermore, spatial cognition and visuospatial ability refer to the surgeons’ ability to locate points in space, determine lines and objects’ orientation, assess location in depth, and process motion, including motion in depth [48]. These factors were related to surgical skills acquisition and had been shown to differ between surgeons according to their experience level [13, 49]. In the Pennington et al. study, higher COR medialization on the non-dominant side was explained by the fact that the surgeon led acetabular preparation with the non-dominant hand, where more force and pressure could be exerted while using the power reamers [35].

The current review has some inherent limitations, which should be considered when interpreting the results. First, only two articles reported the functional outcomes; furthermore, these needed to be more consistent regarding when these were reported. Second, the long-term revision rates, mainly secondary to loosening, were not reported, which could be closely related to implant positioning. Third, all the surgeons included were experienced and high-volume, which guards against generalizing the results over low-volume and less experienced surgeons. Fourth, most included implants were cementless, further questioning the results’ applicability over cemented acetabular cups. Fifth, none of the articles accurately described their manual freehand acetabular cup placement technique, which could differ between surgeons. Sixth, the Lwenniek safe zone numbers range was considered by some authors of the included articles to determine the appropriateness of acetabular cup placement; however, the reliability of these numbers has been questioned in the literature and leaning toward more personalized cup placements safe target zones as well as considering the combined anteversion are recommended. Finally, although all studies were comparative, all were retrospective.

## Conclusion

Surgeon handedness and whether he or she is operating on the dominant or non-dominant side could affect the acetabular cup positioning during primary THAs regardless of the surgical approach, even in the hands of experienced, high-volume surgeons. Although the functional outcomes were affected, more comparative studies are needed to support this result. Furthermore, the effect of a surgeon’s handedness when young, less experienced, and low-volume surgeons operate needs further evaluation. Likewise, whether the results we obtained in the current review could differ when operating on complex primary or revision THAs is still to be determined. Additionally, the results obtained from the current systematic review should be cautiously interpreted, considering the small number of included articles and their relatively lower quality.

## Electronic supplementary material

Below is the link to the electronic supplementary material.

Supplementary Material 1: PRISMA 2020 Checklist.

Supplementary Material 2: Details of the search strategy.

Supplementary Material 3: Risk of bias and quality assessment using the NIH quality assessment tool for observational cohort and cross sectional studies.

## Data Availability

All the data related to the study are mentioned in the manuscript; however, the raw data are available with the corresponding author and will be provided upon a written request.

## References

[1] Sabharwal S, MacKenzie JS, Sterling RS, Ficke JR, LaPorte DM. Left-handedness among Orthopaedic surgeons and trainees. JB JS Open Access. 2020;5(2). 10.2106/JBJS.OA.20.00019. 10.2106/JBJS.OA.20.00019 PMC7418909 32832824

[2] Brooks NE, Lipman JM, French JC. The right way to teach lefties - exploring the experiences of Left-Handed trainees and surgeons. J Surg Educ. 2023;80(11):1552–66. 10.1016/j.jsurg.2023.07.014. 37563001 10.1016/j.jsurg.2023.07.014

[3] Nagaraj MB, AbdelFattah KR, Farr DE. Laparoscopic ambidexterity in Left-Handed trainees. J Surg Res. 2022;275:203–7. 10.1016/j.jss.2022.02.003. 35305486 10.1016/j.jss.2022.02.003

[4] Savetsky IL, Cammarata MJ, Kantar RS, Diaz-Siso JR, Avashia YJ, Rohrich RJ, Saadeh PB. The left-handed plastic surgery trainee: perspectives and recommendations. Plast Reconstr Surg Glob Open. 2020;8(5):e2686. 10.1097/GOX.0000000000002686. 33133882 10.1097/GOX.0000000000002686 PMC7572112

[5] Cao Z, Liu Y, Yang M, Zhang Z, Kong X, Chai W. Effects of Surgeon Handedness on the outcomes of Unicompartmental knee arthroplasty: a single Center’s experience. Orthop Surg. 2022;14(12):3293–9. 10.1111/os.13549. 36281639 10.1111/os.13549 PMC9732585

[6] Mehta S, Lotke PA. Impact of surgeon handedness and laterality on outcomes of total knee arthroplasties: should right-handed surgeons do only right TKAs? Am J Orthop (Belle Mead NJ). 2007;36(10):530–3. 18033564

[7] Liu L, Zhao F, Zha G, Zheng X, Yang G, Xu S. [Effect of surgeon’s handedness on distribution of prosthesis during primary total knee arthroplasty]. Zhongguo Xiu Fu Chong Jian Wai Ke Za Zhi. 2020;34(6):696–701. 10.7507/1002-1892.201911042. PMID: 32538558; PMCID: PMC8171536. 32538558 10.7507/1002-1892.201911042 PMC8171536

[8] Mcsp ICB, Dipcot JA. A comparison of Dominant and non-dominant hand function in both right- and left-handed individuals using the Southampton Hand Assessment Procedure (SHAP). Br J Hand Therapy. 2016;8(1):4–10. 10.1177/175899830300800101.

[9] Kim J-S, Lee S-G, Park S-K, Lee S-M, Kim B-K, Choi J-H, Kim S-H. Comparison of grip and pinch strength between Dominant and non-dominant hand according to Type of Handedness of Female College Students. J Int Acad Phys Therapy Res. 2011;2(1):201–6.

[10] Sertel M, ŞAHAN TY, Bezgin S, ORAL MA, KOCAMAN AA, ARSLAN SA, Demirci C. OKTAŞ B A comparison of the muscle activation, proprioception and anthropometric characteristics of the Dominant and non-dominant wrists. J Basic Clin Health Sci 6 (1):25–32.

[11] Jee H, Park J. Comparative analyses of the Dominant and Non-dominant Upper limbs during the abduction and adduction motions. Iran J Public Health. 2019;48(10):1768–76. 31850253 PMC6908899

[12] McGrath TM, Waddington G, Scarvell JM, Ball NB, Creer R, Woods K, Smith D. The effect of limb dominance on lower limb functional performance–a systematic review. J Sports Sci. 2016;34(4):289–302. 10.1080/02640414.2015.1050601. 26055387 10.1080/02640414.2015.1050601

[13] Vajsbaher T, Schultheis H, Sa-ngasoongsong P, Watcharopas R, Yin MS, Haddawy P, Center BSC. The Role of Spatial Cognition in Surgical Navigation in Arthroscopic Surgery.

[14] Moloney D, Bishay M, Ivory J, Pozo J. Failure of the sliding hip screw in the treatment of femoral neck fractures: ‘left-handed surgeons for left-sided hips’. Injury 25 Suppl. 1994;2B9–13. 10.1016/0020-1383(94)90194-5. 10.1016/0020-1383(94)90194-5 7960081

[15] Kim SC, Lim YW, Kwon SY, Jo WL, Ju SH, Park CJ, Lee CW, Kim YS. Level of surgical experience is associated with change in hip center of rotation following cementless total hip arthroplasty: a radiographic assessment. PLoS ONE. 2017;12(5):e0178300. 10.1371/journal.pone.0178300. 28542504 10.1371/journal.pone.0178300 PMC5443567

[16] Wan Z, Boutary M, Dorr LD. The influence of acetabular component position on wear in total hip arthroplasty. J Arthroplasty. 2008;23(1):51–6. 10.1016/j.arth.2007.06.008. 18165028 10.1016/j.arth.2007.06.008

[17] Kennedy JG, Rogers WB, Soffe KE, Sullivan RJ, Griffen DG, Sheehan LJ. Effect of acetabular component orientation on recurrent dislocation, pelvic osteolysis, polyethylene wear, and component migration. J Arthroplasty. 1998;13(5):530–4. 10.1016/s0883-5403(98)90052-3. 9726318 10.1016/s0883-5403(98)90052-3

[18] Meermans G, Grammatopoulos G, Innmann M, Beverland D. Cup placement in primary total hip arthroplasty: how to get it right without navigation or robotics. EFORT Open Rev. 2022;7(6):365–74. 10.1530/EOR-22-0025. 35638598 10.1530/EOR-22-0025 PMC9257731

[19] Grammatopoulos G, Gofton W, Cochran M, Dobransky J, Carli A, Abdelbary H, Gill HS, Beaule PE. Pelvic positioning in the supine position leads to more consistent orientation of the acetabular component after total hip arthroplasty. Bone Joint J. 2018;100–B(10):1280–8. 10.1302/0301-620X.100B10.BJJ-2018-0134.R1. 30295537 10.1302/0301-620X.100B10.BJJ-2018-0134.R1

[20] Takada R, Jinno T, Miyatake K, Hirao M, Yagishita K, Yoshii T, Okawa A. Supine versus lateral position for accurate positioning of acetabular cup in total hip arthroplasty using the modified Watson-Jones approach: a randomized single-blind controlled trial. Orthop Traumatol Surg Res. 2019;105(5):915–22. 10.1016/j.otsr.2019.05.004. 31204181 10.1016/j.otsr.2019.05.004

[21] Christensen TH, Egol A, Pope C, Shatkin M, Schwarzkopf R, Davidovitch RI, Aggarwal VK. How does Surgical Approach affect characteristics of dislocation after primary total hip arthroplasty? J Arthroplasty. 2023;38(Suppl 2):S300–5. 10.1016/j.arth.2023.05.034. 37236286 10.1016/j.arth.2023.05.034

[22] Ramadanov N, Ostojic M, Lazaru P, Liu K, Hable R, Marinova-Kichikova P, Dimitrov D, Becker R. Risk factors and predictors for functional outcome and complication rate in total hip arthroplasty through minimally invasive and conventional approaches: a systematic review and Meta-regression analysis of 41 randomized controlled trials. J Clin Med. 2023;12(18):5895. 37762836 10.3390/jcm12185895 PMC10531834

[23] Bosker BH, Verheyen CC, Horstmann WG, Tulp NJ. Poor accuracy of freehand cup positioning during total hip arthroplasty. Arch Orthop Trauma Surg. 2007;127(5):375–9. 10.1007/s00402-007-0294-y. 17297597 10.1007/s00402-007-0294-y PMC1914284

[24] Hoskins W, Rainbird S, Lorimer M, Graves SE, Bingham R. What can we learn from surgeons who perform THA and TKA and have the lowest revision rates? A study from the Australian Orthopaedic Association National Joint Replacement Registry. Clin Orthop Relat Res. 2022;480(3):464–81. 10.1097/CORR.0000000000002007. 34677162 10.1097/CORR.0000000000002007 PMC8846272

[25] Khalifa AA, Abdelnasser MK, Ahmed AM, Shetty GM, Abdelaal AM. Smartphone application helps improve the Accuracy of Cup Placement by Young, less-experienced surgeons during primary total hip arthroplasty. Arch Bone Jt Surg. 2022;10(3):278–85. 10.22038/abjs.2021.52402.2587. 35514765 10.22038/ABJS.2021.52402.2587 PMC9034791

[26] Yang G, Li Y, Zhang H. The influence of pelvic tilt on the Anteversion Angle of the Acetabular Prosthesis. Orthop Surg. 2019;11(5):762–9. 10.1111/os.12543. 31663281 10.1111/os.12543 PMC6819173

[27] Eftekhary N, Shimmin A, Lazennec JY, Buckland A, Schwarzkopf R, Dorr LD, Mayman D, Padgett D, Vigdorchik J. A systematic approach to the hip-spine relationship and its applications to total hip arthroplasty. Bone Joint J. 2019;101–B(7):808–16. 10.1302/0301-620X.101B7.BJJ-2018-1188.R1. 31256658 10.1302/0301-620X.101B7.BJJ-2018-1188.R1

[28] Luu K, Nishioka ST, Lawton DRY, Unebasami E, Andrews SN, Nakasone CK. Influence of obesity and intra-operative imaging guidance technology on acetabular cup positioning in total hip arthroplasty. Arch Orthop Trauma Surg. 2023;143(11):6857–63. 10.1007/s00402-023-04922-x. 37270739 10.1007/s00402-023-04922-x

[29] Abdel MP, von Roth P, Jennings MT, Hanssen AD, Pagnano MW. What safe zone? The vast majority of dislocated THAs are within the Lewinnek Safe Zone for Acetabular component position. Clin Orthop Relat Res. 2016;474(2):386–91. 10.1007/s11999-015-4432-5. 26150264 10.1007/s11999-015-4432-5 PMC4709312

[30] Lewinnek GE, Lewis JL, Tarr R, Compere CL, Zimmerman JR. Dislocations after total hip-replacement arthroplasties. J Bone Joint Surg Am. 1978;60(2):217–20. 641088

[31] Callanan MC, Jarrett B, Bragdon CR, Zurakowski D, Rubash HE, Freiberg AA, Malchau H. The John Charnley Award: risk factors for cup malpositioning: quality improvement through a joint registry at a tertiary hospital. Clin Orthop Relat Res. 2011;469(2):319–29. 10.1007/s11999-010-1487-1. 20717858 10.1007/s11999-010-1487-1 PMC3018230

[32] van Erp JHJ, Snijders TE, Weinans H, Castelein RM, Schlosser TPC, de Gast A. The role of the femoral component orientation on dislocations in THA: a systematic review. Arch Orthop Trauma Surg. 2022;142(6):1253–64. 10.1007/s00402-021-03982-1. 34101017 10.1007/s00402-021-03982-1 PMC9110501

[33] Page MJ, McKenzie JE, Bossuyt PM, Boutron I, Hoffmann TC, Mulrow CD, Shamseer L, Tetzlaff JM, Akl EA, Brennan SE, Chou R, Glanville J, Grimshaw JM, Hrobjartsson A, Lalu MM, Li T, Loder EW, Mayo-Wilson E, McDonald S, McGuinness LA, Stewart LA, Thomas J, Tricco AC, Welch VA, Whiting P, Moher D. The PRISMA 2020 statement: an updated guideline for reporting systematic reviews. Syst Rev. 2021;10(1):89. 10.1186/s13643-021-01626-4. 33781348 10.1186/s13643-021-01626-4 PMC8008539

[34] National Institutes of Health. (2014). Quality Assessment Tool for Observational Cohort and Cross-Sectional Studies. Available online at: https://www.nhlbi.nih.gov/health-pro/guidelines/in-develop/cardiovascular-risk-reduction/tools/cohort. Accessed 1-10-2023.

[35] Pennington N, Redmond A, Stewart T, Stone M. The impact of surgeon handedness in total hip replacement. Ann R Coll Surg Engl. 2014;96(6):437–41. 10.1308/003588414X13946184902488. 25198975 10.1308/003588414X13946184902488 PMC4474195

[36] Song X, Ni M, Li H, Li X, Li X, Fu J, Chen J. Is the cup orientation different in bilateral total hip arthroplasty with right-handed surgeons using posterolateral approach? J Orthop Surg Res. 2018;13(1):123. 10.1186/s13018-018-0789-y. 29792206 10.1186/s13018-018-0789-y PMC5967059

[37] Kong X, Yang M, Li X, Ni M, Zhang G, Chen J, Chai W. Impact of surgeon handedness in manual and robot-assisted total hip arthroplasty. J Orthop Surg Res. 2020;15(1):159. 10.1186/s13018-020-01671-0. 32316973 10.1186/s13018-020-01671-0 PMC7171772

[38] Kong X, Yang M, Ong A, Guo R, Chen J, Wang Y, Chai W. A surgeon’s handedness in direct anterior approach-hip replacement. BMC Musculoskelet Disord. 2020;21(1):516. 10.1186/s12891-020-03545-2. 32746833 10.1186/s12891-020-03545-2 PMC7397678

[39] Learmonth ID, Young C, Rorabeck C. The operation of the century: total hip replacement. Lancet. 2007;370(9597):1508–19. 10.1016/S0140-6736(07)60457-7. 17964352 10.1016/S0140-6736(07)60457-7

[40] Minoda Y, Kadowaki T, Kim M. Acetabular component orientation in 834 total hip arthroplasties using a manual technique. Clin Orthop Relat Res. 2006;445:186–91. 10.1097/01.blo.0000201165.82690.f8. 16467620 10.1097/01.blo.0000201165.82690.f8

[41] Crawford DA, Adams JB, Hobbs GR, Lombardi AJV Jr., Berend KR. Surgical Approach and Hip Laterality affect accuracy of Acetabular Component Placement in primary total hip arthroplasty. Surg Technol Int. 2019;35:377–85. 31524283

[42] Grammatopoulos G, Alvand A, Monk AP, Mellon S, Pandit H, Rees J, Gill HS, Murray DW. Surgeons’ accuracy in achieving their desired Acetabular Component Orientation. J Bone Joint Surg Am. 2016;98(17):e72. 10.2106/JBJS.15.01080. 27605697 10.2106/JBJS.15.01080

[43] Fujita K, Kabata T, Maeda T, Kajino Y, Iwai S, Kuroda K, Hasegawa K, Tsuchiya H. The use of the transverse acetabular ligament in total hip replacement: an analysis of the orientation of the trial acetabular component using a navigation system. Bone Joint J. 2014;396–B. 10.1302/0301-620X.96B3.32726. 10.1302/0301-620X.96B3.32726 24589783

[44] Rutherford M, O’Connor JD, Hill JC, Beverland DE, Lennon AB, Dunne NJ. Patient positioning and cup orientation during total hip arthroplasty: assessment of current UK practice. Hip Int. 2019;29(1):89–95. 10.1177/1120700018760818. 29783888 10.1177/1120700018760818

[45] Parratte S, Argenson JN. Validation and usefulness of a computer-assisted cup-positioning system in total hip arthroplasty. A prospective, randomized, controlled study. J Bone Joint Surg Am. 2007;89(3):494–9. 10.2106/JBJS.F.00529. 17332097 10.2106/JBJS.F.00529

[46] Dorr LD, Malik A, Wan Z, Long WT, Harris M. Precision and bias of imageless computer navigation and surgeon estimates for acetabular component position. Clin Orthop Relat Res. 2007;465:92–9. 10.1097/BLO.0b013e3181560c51. 17693877 10.1097/BLO.0b013e3181560c51

[47] Barrack RL, Krempec JA, Clohisy JC, McDonald DJ, Ricci WM, Ruh EL, Nunley RM. Accuracy of acetabular component position in hip arthroplasty. J Bone Joint Surg Am. 2013;95(19):1760–8. 10.2106/JBJS.L.01704. 24088968 10.2106/JBJS.L.01704

[48] Vasilyeva M, Lourenco SF. Development of spatial cognition. Wiley Interdiscip Rev Cogn Sci. 2012;3(3):349–62. 10.1002/wcs.1171. 26301467 10.1002/wcs.1171

[49] Vajsbaher T, Schultheis H, Francis NK. Spatial cognition in minimally invasive surgery: a systematic review. BMC Surg. 2018;18(1):94. 10.1186/s12893-018-0416-1. 30404634 10.1186/s12893-018-0416-1 PMC6223063

