# Supplementary material for: Surgeon handedness affects the acetabular cup positioning during primary total hip arthroplasty regardless of the surgical approach. a systematic review and metanalysis
Source: BMC Musculoskelet Disord. 2024 Oct 7;25:792. doi: 10.1186/s12891-024-07868-2 (PMC11460204; doi:10.1186/s12891-024-07868-2)
Supplement: Supplementary file 2 — Supplementary Material 2: Details of the search strategy. [file 12891_2024_7868_MOESM2_ESM.pdf]

Three databases (PubMed, Embase, and Scopus) were searched for eligible articles.

The search strategy was built following the PICO format:

1. Population (P): Patients undergoing total hip arthroplasty (THA) where we utilized the following MeSH term: “Replacement, Hip” “arthroplasty” and text words: “Total Hip Arthroplasty" “THA" “Hip Replacement" “Hip Arthroplasty".

2. Intervention (I): Surgery performed on the dominant side (e.g., right THA for right-handed surgeons and vice versa).

3. Comparison (C): Surgery performed on the non-dominant side (e.g., left THA for right-handed surgeons and vice versa).

For both 2 and 3 we utilized the following: MeSH term: handedness, and the text words: “Surgeon handedness" “Hand dominance" “Left-handed surgeon” and “Right-handed surgeon”

4. Outcome (O): Acetabular cup positioning (Abduction (inclination) angle, and anteversion) where the following search text words were utilized “Acetabular cup positioning" “Acetabular component alignment" “Acetabular cup placement" “Inclination" “Abduction angle" “Anteversion”

We started a crude search limited to English literature published in the past 20 years using the following simple text words “Total hip and handedness” to capture as many articles as possible. Then, we tried the following detailed search strategy combinations:

PubMed Search Strategy:

A: (("Surgeon handedness"[Title/Abstract] OR "Hand dominance"[Title/Abstract] OR "Left-handed surgeon"[Title/Abstract] OR "Right-handed surgeon"[Title/Abstract]) AND ("Acetabular cup positioning"[Title/Abstract] OR "Acetabular component alignment"[Title/Abstract] OR "Acetabular cup placement"[Title/Abstract]) AND ("Total Hip Arthroplasty"[Title/Abstract] OR "THA"[Title/Abstract])) AND

B: ((“Total Hip Arthroplasty” OR “THA” OR “Hip Replacement” OR “Hip Arthroplasty”) AND (“Surgeon handedness” [Title/Abstract] OR “Hand dominance” [Title/Abstract] OR “Left-handed surgeon” [Title/Abstract] OR “Right-handed surgeon” [Title/Abstract]) AND (“Acetabular cup positioning” [Title/Abstract] OR “Acetabular component alignment” [Title/Abstract] OR “Acetabular cup placement” [Title/Abstract] OR “Acetabular positioning” [Title/Abstract] OR “Acetabular cup orientation” [Title/Abstract]))

C: (((((replacement, total hip[MeSH Terms]) OR (arthroplasty[MeSH Terms])) OR (total hip arthroplasty[Title/Abstract])) OR (hip arthroplasty[Title/Abstract])) AND (((handedness[MeSH Terms]) OR (Surgeon handedness[Title/Abstract])) OR (Hand dominance[Title/Abstract]))) AND ((((((Acetabular cup positioning[Title/Abstract]) OR (Acetabular component alignment[Title/Abstract])) OR (Acetabular cup placement[Title/Abstract])) OR (cup inclination[Title/Abstract])) OR (cup abduction[Title/Abstract])) OR (cup anteversion[Title/Abstract]))

Scopus Search Strategy:

(TITLE-ABS(“Surgeon handedness” OR “Hand dominance” OR “Left-handed surgeon” OR “Right-handed surgeon”) AND TITLE-ABS(“Acetabular cup positioning” OR “Acetabular component alignment” OR “Acetabular cup placement”) AND TITLE-ABS(“Total Hip Arthroplasty” OR “THA”)) AND PUBYEAR > 2003

Embase Search Strategy through Ovid:

Your Journals@Ovid

Embase <1974 to 2024 August 26>

1	hip arthroplasty.mp. [mp=ti, ab, tx, ct, sh, hw, tn, ot, dm, mf, dv, kf, fx, dq]	55362
2	"total hip*".m_titl.	28238
3	1 or 2	62181
4	handedness.mp. [mp=ti, ab, tx, ct, sh, hw, tn, ot, dm, mf, dv, kf, fx, dq]	20483
5	right handed.m_titl.	543
6	left handed.m_titl.	880
7	surgeon handedness.m_titl.	8
8	4 or 5 or 6 or 7	21252
9	cup inclination.m_titl.	32
10	cup anteversion.m_titl.	45
11	acetabular cup.m_titl.	643
12	cup abduction.m_titl.	9
13	inclination.mp. [mp=ti, ab, tx, ct, sh, hw, tn, ot, dm, mf, dv, kf, fx, dq]	20008
14	anteversion.mp. [mp=ti, ab, tx, ct, sh, hw, tn, ot, dm, mf, dv, kf, fx, dq]	5773
15	2 or 3	62181
16	4 or 5 or 6	21252
17	9 or 10 or 11 or 12 or 13 or 14	24714
18	15 and 16 and 17	23

The maximum number of articles we obtained from the detailed search above was a total of 98 articles, and only four were eligible and could be obtained for qualitative and quantitative results synthesis.

N.B.: The initial search was carried out on May 2023, and the search was repeated on April 2024; no further studies were included.
