# Supplementary material for: Surgeon handedness affects the acetabular cup positioning during primary total hip arthroplasty regardless of the surgical approach. a systematic review and metanalysis
Source: BMC Musculoskelet Disord. 2024 Oct 7;25:792. doi: 10.1186/s12891-024-07868-2 (PMC11460204; doi:10.1186/s12891-024-07868-2)
Supplement: Supplementary file 3 — Supplementary Material 3: Risk of bias and quality assessment using the NIH quality assessment tool for observational cohort and cross sectional studies. [file 12891_2024_7868_MOESM3_ESM.pdf]

Table S1. Studies were assessed using the NIH quality assessment tool for observational cohort and cross sectional studies. **(**https://www.nhlbi.nih.gov/health-topics/study-quality-assessment-tools)

Major Components	Pennington et al.	Song et al.	Kong et al. (PLA)	Kong et al. (DA)
1. Was the research question or objective in this paper clearly stated?	Yes	Yes	Yes	Yes
2. Was the study population clearly specified and defined?	Yes	Yes	Yes	Yes
3. Was the participation rate of eligible persons at least 50%?	NA	NA	NA	NA
4. Were all the subjects selected or recruited from the same or similar populations (including the same time period)? Were inclusion and exclusion criteria for being in the study prespecified and applied uniformly to all participants?	CD	CD	CD	CD
5. Was a sample size justification, power description, or variance and effect estimates provided?	No	No	No	No
6. For the analyses in this paper, were the exposure(s) of interest measured prior to the outcome(s) being measured?	Yes	Yes	Yes	Yes
7. Was the timeframe sufficient so that one could reasonably expect to see an association between exposure and outcome if it existed?	Yes	Yes	Yes	Yes
8. For exposures that can vary in amount or level, did the study examine different levels of the exposure as related to the outcome (e.g., categories of exposure, or exposure measured as continuous variable)?	NA	NA	NA	NA
9. Were the exposure measures (independent variables) clearly defined, valid, reliable, and implemented consistently across all study participants?	CD	CD	CD	CD
10. Was the exposure(s) assessed more than once over time?	No	No	No	No
11. Were the outcome measures (dependent variables) clearly defined, valid, reliable, and implemented consistently across all study participants?	Yes	Yes	Yes	Yes
12. Were the outcome assessors blinded to the exposure status of participants?	No	No	No	No
13. Was loss to follow-up after baseline 20% or less?	NA	NA	NA	NA
14. Were key potential confounding variables measured and adjusted statistically for their impact on the relationship between exposure(s) and outcome(s)?	No	No	No	No
Quality Rating	Fair (5/14)	Fair (5/14)	Fair (5/14)	Fair (5/14)

CD: Cannot Determine. NA: Not Applicable. NR: Not Reported. Quality was rated as poor (0–4 out of 14 questions), fair (5–10 out of 14 questions), or good (11–14 out of 14 questions).
